# The iceberg of dementia risk: empirical and conceptual arguments in favor of structural interventions for brain health

**DOI:** 10.1016/j.cccb.2023.100193

**Published:** 2023-12-16

**Authors:** Timothy Daly

**Affiliations:** Bioethics Program, FLACSO Argentina, Tucumán 1966, C1050 AAN, Buenos Aires, Argentina; Science Norms Democracy UMR 8011, Sorbonne Université, 1 Rue Victor Cousin 75005, Paris, France

**Keywords:** Public health, Dementia prevention, Brain health justice, Structural interventions

## Abstract

While pharmacological interventions for dementia struggle to demonstrate improved outcomes for patients and at-risk populations, non-pharmacological lifestyle interventions have been proposed as a tool to achieve dementia risk reduction. In this review, it is argued that lifestyle modification alone is a surface-level intervention from the point of view of fair and far-reaching dementia prevention. Below the tip of this “iceberg of dementia risk,” there are living conditions and social structures that represent deeper contributions to risk in the population. It is argued that alongside lifestyle modification, activist research and structural interventions are needed to make our society fairer and more dementia-resilient.

## Highlights

• Non-pharmacological lifestyle interventions have been proposed as a tool to achieve dementia risk reduction.
• Lifestyle modification alone is a surface-level intervention from the point of view of fair and far-reaching dementia prevention. Below the tip of this “iceberg of dementia risk,” there are living conditions and social structures that represent deeper contributions to risk in the population.
• Alongside lifestyle modification, activist research and structural interventions are needed to make our society fairer and more dementia-resilient.

## 1 Introduction: The need for ambitious policy change in dementia prevention

The decades-long quest for pharmacological solutions to the major public health problem of dementia has generally met with frustration. Though symptomatic treatments for cognitive decline exist, and recent results from high-clearance antibodies in patients with Alzheimer's disease show some promise [1], there is a need for ambitious thinking about non-pharmacological solutions for the prevention of later-life amnestic dementia.

However, this review paper on non-pharmacological solutions for dementia is different from a typical review. It will follow a strongly argumentative structure to argue for radical change in public policymaking. To achieve this end, not only will it focus on empirical data, but also on both original and previously published conceptual arguments. It will therefore draw on literature from public health, randomized clinical trials (RCTs), sociology of science, and also ethics and philosophy of science. It is motivated by the intuition from research ethics that the most scientifically valid research is both fair and far-reaching, ie. it has high social value [2].

It will be argued that much (but not all) discourse around dementia prevention has been overly focused on individuals and their lifestyle behaviors in ways that not only limit the potential of fair and far-reaching prevention by making society fairer, but are also overly coercive because they place the burdens of research on individuals (an expectation that they will change their behaviors because of the discovery of modifiable risk factors) without equally distributing the benefits of research [3].

While there are possible ethical justifications for public health policies based on coercive lifestyle modification including “social benefit; by the benefit to the coerced; and by a right of others in society to prevent the self-destructive individual from placing unfair burdens upon them” [4], here, it will be argued that in the context of dementia, there is poor evidential basis for coercion and certainly none for victim blaming [5]. Instead, it will be argued that research into dementia prevention and the policy making it has inspired so far has had unexploited social value because it has been mostly “tip and no iceberg” [6]. That is because dementia risk, from a public health point of view, is likely to resemble the following equation:

Modifiabledementiarisk=lifestylebehaviors+dailyliving,workandleisureconditions+broadereconomic,socialandpoliticalstructures.

Lifestyle behaviors represent the visible “tip” of the iceberg, whereas daily living conditions and sociopolitical structures are below the surface, i.e. are less immediately visible at the level of policymaking [6] and even at the level of what scientists understand as a cause for phenomena [7]. Lifestyle behaviors thus provide a convenient locus for change, but over-reliance on them is ultimately ineffective for most chronic health conditions [8]. In particular, if policymaking for dementia risk reduction focuses only on lifestyle behaviors, and does not highlight the need for changes in broader conditions in which people live, grow and work, then it will actually widen health inequities [9]. Thus, to maximize the social value of research and policymaking around dementia prevention, we must encourage structural interventions within society to make it healthier and fairer. Drawing on evidence and arguments in favor of this is the goal of this review paper.

The structure of this review is as follows. First, the rationale, implementation, and limitations of individual-level non-pharmacological interventions for dementia will be described. Second, the iceberg model of dementia risk will be presented in more depth. Thirdly, the implementation of structural interventions will be discussed. The conclusion will discuss preventive policy, with an emphasis on co-creation of risk-reducing environments, lifestyle and shared responsibility, and the need for new vocabulary to talk about a dementia-resilient society.

## 2 Individual non-pharmacological interventions for dementia prevention: rationale, implementation, and limitations

Presently, over 50 million people live with dementia, and by 2050, this number may be around 150 million [10]. However, there are promising data suggesting that many cases of dementia are preventable. Perhaps the most important finding is that rates of dementia in the United States and Europe are declining by 13% per decade [11]. Furthermore, two commissioned expert panels by the Lancet in 2017 and 2020 found that 40% cases of dementia are associated with 12 modifiable risk factors. These factors include early life (less education), midlife (hearing loss, brain injury, hypertension, alcohol consumption, obesity), and late life (smoking, depression, social isolation, physical inactivity, diabetes, air pollution) [12,13]. Results from China [14,15], Brazil [16], and Australia [17] suggest that in certain populations, the population attributable fraction of modifiable risk due to lifestyle behaviors may be more than 50%. These data are a reminder that we should be as ambitious as possible about dementia prevention.

Crucially, new data suggest that the drop in dementia rates in the West cannot be attributed to people developing less neuropathology in their brain (e.g. amyloid and tau proteins characteristic of Alzheimer's disease) [18]. Instead, it has been suggested that other factors, related to cerebrovascular health (ie. improved health of brain blood vessels), are making more and more people “resilient” to the buildup of brain pathology that would otherwise be quite strongly associated with cognitive decline [19]. In other words, it is likely that ambitious prevention of dementia, from both an individual and public health point of view, will require actions that are “good for the heart and good for the brain” [20]. Furthermore, action against modifiable risk factors to improve cerebrovascular health “could be effective even in those with genetic predisposition to dementia” [21].

Non-pharmacological interventions for dementia are generally understood in the dementia literature as multi-domain (physical, nutritional, cognitive, and social) lifestyle interventions acting against risk factors. Clinical translation is essentially understood as converting promising data into multi-domain interventions to be tested via the randomized controlled trials (RCT) methodology. The most promising data in favor of this interventional approach is the Finnish Geriatric “FINGER” trial.

Launched approximately a decade ago, the FINGER RCT was a 2-year multi-domain physical and cognitive interventional trial with a trial population aged between 60 and 77 that consisted of nutritional guidance; exercise; cognitive training, social activity, and management of metabolic and vascular risk factors versus regular health advice for controls. The trial led to a small composite reduction in cognitive decline versus controls [22]. Following positive reception of these results, particularly against the backdrop of failed clinical trials with pharmacological treatments for dementia, the methodology of FINGER was then exported via the worldwide FINGERS initiative to “prevent cognitive impairment and dementia ... generating high-quality scientific evidence to support public health and clinical decision-making ... [and] support the implementation of preventive strategies and translation of research findings into practice” [23]. However, it is noteworthy that the FINGER trial's positive findings of multi-domain intervention on cognition have not so far been replicated by similar studies. The French Multi-domain Alzheimer's Prevention Trial (“MAPT”) with omega-3 supplementation and lifestyle intervention and the Dutch “Prevention of dementia by intensive vascular care” (PreDIVA) studies did not find significant beneficial effects on cognition of these interventions in populations of similar ages in these different European countries [24,25].

Though the treatment material of these interventions is markedly different from pharmacological trials (ie. a set of behaviors as opposed to drugs), there are many methodological similarities. Not only is their therapeutic value tested via RCT, analyses of these trials focus on dose-response, tolerability, and other aspects of RCT design inherited from drug studies [22,26]. In other words, these multi-domain interventions look like attempts to “package” lifestyle interventions as though they were a kind of pharmacological intervention [27].

This focus on delivering multi-domain interventions to individuals is paradoxical because the most convincing data on falling rates of dementia and modifiable risk factors comes not from the study of individuals but rather from studying populations [11,13]. It is probable that the “lifestyle” lens through which risk is understood, as a convenient locus for action, contributes to the framing of dementia risk as an individual problem [28]. However, it is worth asking to what extent this focus on individuals results from “following the science” or perhaps other forms of political or philosophical bias. Indeed, Carey and colleagues argue that lifestyle solutions to public health problems are “more politically palatable, more immediately relatable to the problem at hand and easier to devise than ‘upstream’ interventions” [8]. Other explanations include the philosophical idea that individuals are morally responsible for maintaining their own health in the context of prevention through control of their own behaviour [29].

And yet it will be the central argument of this review that “upstream” interventions are required for more adequate dementia prevention in ways that maximize all the life-years the population is exposed to dementia risk and protective factors. Given both the sheer extent of risk present in the population, as well as the unequal distribution of exposure to risk and protective factors throughout the lifetime, it is both therapeutically inadequate and ethically problematic to “package” lifestyle interventions as a public health solution to the problem of dementia risk in an aging population [5].

As a reminder of the extent of risk present in the population, over 50 million people are already living with dementia worldwide, and prevalence is set to triple by 2050 [10]. Moreover, it has been argued that over 400 million people worldwide are currently in a high-risk group [30]. While there are genetic risk factors for Alzheimer's and other dementias, in the older population, dementia typically presents as “a diffuse clinical syndrome representing the gradual accumulation of multiple pathologies, arising from multiple interlocking risk factors over the life course” [31]. This led the public health approach present in expert groups commissioned by the Lancet to focus on “all the different types of dementia” [12] rather than an exclusive focus on the most common kind, Alzheimer's disease.

Of those “interlocking risk factors,” 40% of cases of age-related dementia are associated with 12 potentially modifiable risk factors that people are exposed to across the lifetime, centered around less education, poor physical and social health, and air pollution [13]. Risk exposure is important across early life (less education), midlife (hearing loss, brain injury, hypertension, alcohol consumption, obesity), and late life (smoking, depression, social isolation, physical inactivity, diabetes, air pollution). It is for this reason of therapeutic inadequacy — in other words, the “dose” of dementia risk reduction being too low given the extent of risk in the population — that packaging non-pharmacological interventions as relatively short multi-domain interventions as a solution to reduce risk in the population is therapeutically inadequate [9,27]. Results from the FINGER, MAPT, and preDIVA trials are not of sufficient magnitude to affect dementia risk throughout the aging population. Such interventions overlook a lifetime of risk [5], and they also cannot explain falling risk throughout the population. Indeed, though the overall prevalence of dementia is set to triple by 2050 across the world, “dementia incidence has declined in many countries without any widespread adoption of individualised approaches to risk reduction” [32].

Beyond the limited public health impact of multi-domain interventions, over-reliance on individual behavior change may over-emphasize an individual's responsibility for their own health while simultaneously distracting away from the need for action against equalities. Indeed, Horstkötter et al. warn against the dilemma facing public health campaigns that “it is still rather unclear how one can get … prevention without risking … blaming patients” [3]. This risk of blame exists despite the fact that the 2020 Lancet commission states that “little evidence exists for any single specific activity protecting against dementia … [and that] many risk factors cluster around inequalities [in…] minority ethnic groups and in vulnerable populations” and offers ‘Tackle inequality’ as an important policy priority for public health for dementia prevention [13]. There is only weak evidence available, visible only at the level of thousands or more individuals to see a measurable effect, suggesting that lifestyle modification may change dementia risk. In light of this scarce evidence, at most, an individual's only responsibility would be “prudential,” i.e. a duty they have towards themselves to stay in good health if they consider that to be an important goal for their own personal reasons [33].

It is important to think beyond individuals for dementia prevention. There is a burgeoning literature on social health [34] and social determinants of health [35] and their relationship with dementia, raising questions of health justice. Talk about social determinants of health in the context of dementia prevention refers to the ways in which unequal access to certain environmental resources, through the mechanisms of different kinds of deprivation, lead to increased dementia risk [36]. The most important aspect is socioeconomic disparities, which have been studied for decades for dementia and other chronic diseases [37]. Hofbauer and Rodriguez recently validated a social deprivation index in US and European older adults [38]. A recent global study showed that social connectedness and capital promotes cognitive health and reduces dementia risk [39], and Wang et al. concluded a meta-analysis of 39 studies finding low socioeconomic status “substantially increased the risk of dementia” [40]. Bothongo et al. recently found socioeconomic deprivation to be an independent risk factor for dementia, leading to more substantial risk “than for any established modifiable risk factor” in a diverse London population [41].

There are dementia prevention campaigns in dozens of countries, with the prevailing idea that lifestyle action will provide solutions. The idea is that if people are sufficiently informed of the kind of “multi-domain interventions” they need to carry out in their lives to reduce risk, then they themselves can reduce their own risk of developing cognitive decline and dementia. But if public health campaigns only provide messaging without addressing disparate access to resources, they will actually widen health inequalities insofar as they do not address disparate access to resources required for risk reduction [9]. Other problematic aspects of over-reliance on multi-domain intervention studies are the problem of healthy volunteer bias [9] and people with lower income being less likely to take part in such studies [42].

Finally, the “lifestyle” framing of non-pharmacological intervention assumes that risk reduction takes place in an environmental void. While over-emphasizing lifestyle in ways that put a moral onus on individuals to change their behaviors [29], it simultaneously distracts away from the contribution of air pollution and other environmental stressors [13]. For example, a recent meta-analysis found that lifetime exposure to <2.5 micron particulate matter is robustly associated with risk of later dementia [43]. In residential areas, increased exposure to traffic noise increases dementia risk [44], which could also partly explain why hearing loss is associated with dementia risk [13], even though establishing causal relationships is difficult for these kinds of epidemiological studies. Just encouraging citizens to undertake lifestyle behavioral change—which may encourage them to, for example, exercise in dangerous, polluted environments—does not encourage health equity, and in fact pushes it ever further away.

In summary, the position presented here in this section has been that understanding dementia risk and interventions aiming to reduce it through the lens of lifestyle and multi-domain interventions does not go far enough to allow for efficient and equitable risk reduction throughout the population. The next section will present the argument that if living and working environments and societal structures play a crucial role in dementia risk, then it makes sense to change them in ways that favor brain health, so as to increase the probability that dementia risk reduction will be fair and far-reaching.

## 3 The iceberg of modifiable dementia risk

Much language used to talk about science is metaphorical, ie. based on the use of images to convey an idea about the structure and causes of a phenomenon [45]. Other metaphors about dementia have included a “tsunami” [46] of dementia risk, and also from gardening, where individuals are understood as plants growing in soil, with the latter being a metaphor for living and working conditions as a reference to social and structural determinants of brain health [5].

**Fig. 1:**
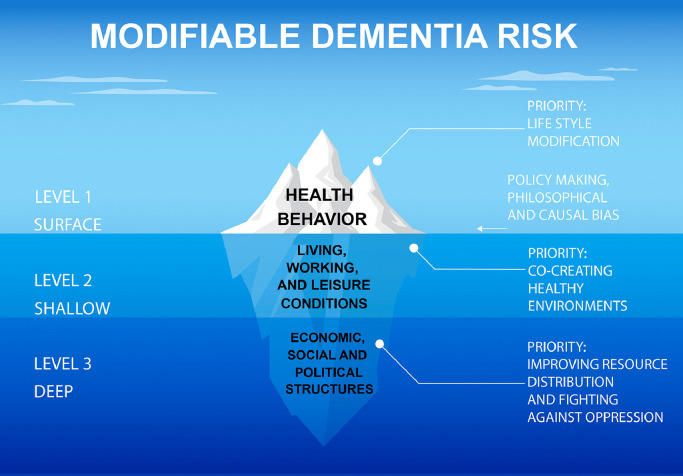
The iceberg of modifiable dementia risk, which invites below-surface conceptualization of dementia risk. It is argued that lifestyle behavior modification represents a “surface” level intervention, whereas improving “conditions” are shallow interventions, and structural interventions are “deep” changes within the sociopolitical order. Truly effective dementia prevention will require interventions across these three levels. This figure is based on the following sources: Baum (2009), and the Figure “The Health Promotion Iceberg” from the following web-page, “What Is Health Promotion”. https://health-inequalities.eu/financing-e-guide/what-is-health-promotion/. Accessed 10-02-2023.

The “iceberg” presented here is metaphorical, and its use is “heuristic,” i.e. is meant to encourage useful and original thinking about certain aspects of a phenomenon without claiming to have exhausted an understanding of its intrinsic nature [47]. The fundamentally-defining feature of the “iceberg” metaphor in the context of public health is how it encourages us to consider that fair and far-reaching policy for health requires “looking below the surface for solutions to improve health” [6], just as an iceberg contains a “tip” (visible part) and a “bummock” (submerged ice) — see Fig. 1.

What ultimately defines the water “surface” is a set of biases about priorities in preventive policy making. There are likely different kinds of short-sighted bias: within policy making itself, in our capitalistic societies that views health as an extension of the idea that health is a kind of individual property and therefore an individual problem [29,48]. However, it may also be related to over-reliance on the drug development view of “translation” in dementia research, in which like other domains within the physical and life sciences, there is a “preference for reductive explanations” that makes it difficult to comprehend and study how “resources and material conditions” play a causal role in complex phenomena like disease risk [7].

When considering the two submerged levels of the iceberg, living, working and leisure “conditions” are shallower and refer to the more immediate environment than sociopolitical structures. Initiatives like the World Health Organization's Healthy Cities approach, instigated in 1977, are grounded in the robust observation between environments and health outcomes [49]. When we talk about the need for more parks, green space, and less pollution, we are invoking this level of policy change.

However, the deeper level of structural causes includes different kinds of oppression [50] including racism [51] and those health inequities imposed by capitalist societies [52] and other kinds of political regimes [53]. Within capitalism, an increasingly important concept is the role played by the private sector in shaping health outcomes. These are known as the commercial determinants of health and they describe the way in which “market fundamentalism and increasingly powerful transnational corporations” have led to a major cause of death and ill health over recent decades [54]. Work from social science suggests that these commercial determinants, including mass consumerism, have deep impacts on health outcomes across the globe [55]. They are increasingly recognized as being relevant to dementia and brain health [56].

Importantly, the barriers between the layers of the model are not watertight. For instance, progress has been delayed in the Healthy Cities initiative by spatial constraints affected by “market forces” [57], widening the spatial dimension of health inequities in both urban and rural contexts [58]. Progress in the Healthy Cities is highly variable between countries [59] and continents [60] due to the convergence and divergence of different societal forces.

Moreover, there is an important asymmetry in the iceberg model. Deep reforms within the structure of society are likely to cause changes at the surface in health behaviors, for example by increasing the amount of leisure across different social strata, associated with lower dementia risk [61]. Conversely, lifestyle modification itself will not penetrate deep enough to change social gradients of health [62,63]. Indeed, data from Japan suggest that lifestyle activities “play a minimal role as mediators between low socio-economic status and dementia” [64]. Thus, tackling those deeper levels of dementia risk, despite their relative invisibility and the long-term logic it would require, emerges as a priority for effective dementia prevention.

## 4 Implementing structural interventions for dementia risk reduction

Deep change is required for dementia prevention to be fair and far-reaching. This will entail “structural interventions,” which are “public health interventions that promote health by altering the structural context within which health is produced and reproduced” [65].

The gist of the argument presented here is that we ultimately need to address what counts as “modifiable” when we discuss action against “modifiable risk factors” in the context of dementia [69]. I will argue that we should be thinking long-term and re-designing society in ways that emulate the falling risks at a population level by maximizing brain and bodily health across the lifetime (Table 1). In other words, the essence of our non-pharmacological interventions should be a widespread and lasting form of “environmental enrichment” across society that gives more universal access to safe and stimulating environments in which risk reduction can take place [70].

**Table 1:** Surface, shallow, and deep public health interventions for dementia prevention along with equity considerations.

Level	Priority for dementia risk reduction	Concrete possibilities for preventive policy	Equity considerations
1: Surface	Lifestyle modification	National campaigns to promote healthy lifestyles Diverse recruitment for RCTs of multi-domain interventions	Respect the “no ought without support” principle in which lifestyle advice is accompanied by actions and resources to make lifestyle change feasible [36].
2: Shallow	Changing daily living conditions	Co-creation of healthy environments with stakeholders: Widespread access to education, green spaces like parks, stimulating forms of intellectual and physical culture Campaigns to promote social connectedness throughout the lifespan	The need for inclusive research (both qualitative and quantitative) to avoid injustice against vulnerable members of society and promote trust in research [66].
3: Deep	Improving resource distribution and fighting against oppression	Overcoming structural barriers to education and work for women, gender and ethnic minorities across the globe Reducing the influence of commercial determinants of health (e.g. advertising)	Decolonising global health [67], i.e. overcoming power inequities in relationships between different countries and health bodies, through a “glocal” approach to global health promotion [68].

At the individual level, environmental enrichment in the form of increased sensory and sensorimotor interactions with the environment has a very positive impact on the brain [71]. Furthermore, such positive impacts can be seen not only during brain development but also in the maintenance of brain plasticity in adult life [72]. There are phylogenetic, neuropathological, and observational data in favor of the notion that “activation of neurons may have a beneficial effect on neuronal function and survival during aging” [73] in ways that are protective against dementia and in line with a “use it or lose it” hypothesis [73,74]. If we could export these positive benefits on individuals to the population level in ways that also address brain health disparities, then great impacts could be had on dementia risk at the population level.

Walsh and colleagues have argued for such interventions in dementia prevention [9,75]. They define their “population-level approach” to dementia prevention as broad measures “with the aim of changing the social, physical, economic, or legislative environments to make them less conducive to the development or maintenance of dementia and its modifiable life course risk factors” [9]. What remains to be implemented is deciding between the prioritization of these dimensions, and above all a method for resolving conflicts between approaches, particularly in a context where resources may be limited and only certain aspects of environments (“social, physical, economic, or legislative”) may be changed.

In any case, such interventions will likely take many different forms and will require adapting to different contexts worldwide [68,76,77]. From a health justice point of view, it is essential that we identify both global and local pre-existing inequalities so as to prioritize action against them in the name of health equity. For instance, the GBD study [10] predicts that women with dementia will continue to outnumber men in 2050, and in the two regions set to see the largest projected dementia increases–the Middle East and eastern sub-Saharan Africa–men and women still have strongly disparate lifetime access to education and work in safe and stimulating environments. Improving access to education for girls across the world would represent a particularly important form of environmental enrichment for women, requiring global efforts.

However, there are other challenges, including the production and evaluation of new kinds of evidence. As Walsh and colleagues mention, “the RCT design is not well suited to evaluating population-based approaches to dementia risk reduction” [78]. As possible alternatives, they suggest the utility of cluster trials, modeling studies, and natural experiment studies to infer causality and “to build the evidence base for population-based dementia risk reduction” [78]. This is particularly important given the limited resources available for research into, and action against, dementia, begging the question of how to distribute research efforts into pharmacological and non-pharmacological solutions in the fairest and most effective ways [5]. Moreover, some aspects of lifestyle advice given to reduce dementia risk over the past decade may not have been entirely evidence-based. This is due to gaps in the scientific literature on dementia risk and health behaviors during midlife [79], despite the fact that this age group is the most-targeted for lifestyle campaigns in different countries [3].

A recent thought-provoking essay by Agustín Ibáñez and colleagues [37] on socioeconomic disparities raises the question of what we prioritize in dementia research. The authors rightly recognise “the biological ripple effects” of disparities and encourage “a multilevel, systematic shift towards diversity through the lens of socioeconomic disparities” to ultimately improve interventions for brain health. While I agree with their call for further research on disparities and their integration with biological models of risk, their use of the term “ripple effect” is reminiscent of an analogy with the “amyloid cascade hypothesis” of Alzheimer's disease. For if we are truly concerned with “addressing the gaps between socioeconomic disparities and biological models of dementia” [37], then we might learn from the fact that those biological models encourage intervention in the processes considered to play a causal role in dementia. This requires seeing hypotheses as tools, whose elaboration should not be thought to replace actual intervention in the deprivation “cascade” [80] rather than simply encourage further study of it.

Indeed, for all the theoretical work spent defending permutations of the amyloid hypothesis, defenders of it have vocally promoted the amyloid-lowering therapeutic strategy as the *raison d’être* of the study of amyloid. In 2006, Sir John Hardy wrote that “the amyloid hypothesis cannot be proved, only disproved: and it will only have been truly useful if it leads to the development of a treatment based on its therapeutic implementation” [81]. This highlights the practical orientation of the biomedical hypothesis as a theoretical tool with therapeutic finality. This practically-oriented thinking has not fully penetrated into work on dementia's relationship with socioeconomic disparities, despite decades of studies on it. For instance, deprivation is not mentioned explicitly as a risk factor in any current dementia prevention guidelines promoted by governmental policy makers [36]. Calls for the need for action against deprivations have been comparatively recent. The Lancet 2020 commission on dementia offers “tackle inequalities” [13] as a priority for dementia prevention and further commissions will probably move in this direction. As previously mentioned, a recent population-level approach to dementia risk reduction, more satisfying than individual-level interventions from a health equity point of view, is being implemented [9,75]. But active calls to make society fairer on the part of dementia researchers are not mainstream; much scientific and journalistic discourse continues to focus on individuals and their lifestyle behaviors [29].

Against this backdrop, it might be tempting to distinguish research, aimed at producing more generalizable knowledge, from activism, acting on the knowledge that we already have. However, between “pure” academic research on one side and “pure” activism on the other, we find “activist research,” which

“*aims at challenging inequality by empowering the powerless, exposing the inequities of the status quo, and promoting social changes that equalize the distribution of resources. Such research is "for" relatively powerless groups, and often involves close social ties and cooperation with these groups*” [82].

Within dementia research, there is much greater scope for studying the needs of people experiencing deprivation and oppression up close, and arguing for structural changes within society to make it fairer and healthier. Qualitative research has revealed that many disadvantaged people perceive “a clear hierarchy of influences” rather than factors “typically listed on par” in academic research [80]. For example, in a sample of low-income African-American men, poverty and unemployment were considered the most important preoccupations that act synergistically to affect health behaviors and produce worse health outcomes [80].

So as to promote truly democratic solutions, different stakeholders should be involved so as to favor the co-creation of risk-reducing environments. The stakeholder survey has been used in work from the Global Brain Health Institute to obtain feedback from residents on the kinds of changes they would like to see to environments — ranging from access to public toilets to campaigns against loneliness — to make them more adapted to the goal of risk-reduction [83]. The themes that emerged from this survey were centered around inclusion, accessibility, and safety. It is vital that we encourage further qualitative research so that questions of “what matters most” to the aging population from a risk reduction point of view can be ascertained [84]. Rosenberg and colleagues showed that rigorous qualitative research can be undertaken while citizens participate in other studies, like those aimed at prevention [85]. However, there is still major stigma around conversations about dementia in society, and even within research [86]. There is a major unmet need in the dementia literature on qualitative studies with disadvantaged people who experience many barriers to participation in research, including risk reduction [42]. We must make every effort for research to be as inclusive as possible [66] through different kinds of layperson co-research [87] and democratic deliberation. In the spirit of inclusive democratic debate, we must be vigilant about overcoming different forms of testimonial injustice, within wider society and also patient organizations, towards minorities as well as people who already have dementia or are at higher risk of developing it [88].

While we continue to study disparities and dementia, researchers should be as explicit as possible about the need for action against deprivation in our unfair societies characterized by social health gradients [36]. Taking the example of climate change, "Our house is on fire and we're looking the other way" (*notre maison brûle et nous regardons ailleurs*) was uttered on September 2nd, 2002 by Jacques Chirac, the then President of the French Republic, as he opened his speech to the plenary assembly of the Fourth Earth Summit in Johannesburg, South Africa. A similar sentiment was taken up by Greta Thunberg with her declaration that “Our house is on fire” at the World Economic Forum in 2019. In dementia research, it is not so much that we look the other way; but rather, we have not fully integrated the sentiment behind a recent expert consensus panel on climate change for research-informed activism: “now that we know, we must act” [89].

## Conclusion: Activist research and structural interventions in a dementia-resilient society

Thanks to theoretical toolkits such as those put forward by Ibáñez and colleagues [37] as well as other researchers [90], researchers who study deprivation and dementia are uniquely positioned to stress the need for evidence-based action against disparities in the struggle for a fairer and healthier society as a form of activist research [82]. And this “fairer and healthier” society is, ultimately, a "dementia-resilient" society [91]. Indeed, we should draw on the success of concepts like “dementia friendly” and associated terms [92] so as to re-invent our language to talk about dementia, and shift away from an exclusive language of individual risk, to framing dementia in terms of systemic and structural determinants [93]. However, we should be sure to communicate expert understanding of dementia risk in ways that make sense to policy makers and other members of society [94]. For instance, even the term “resilience” has important ethical and social dimensions [95]: while generally considered as a property of individuals, we must do our utmost to make sure that resilience to conditions like dementia is as universal as possible in a dementia-resilient society.

The arguments presented here are in line with the prescient work by anthropologist Margaret Lock, who argued a decade ago that dementia and aging were "entangled" [96]. She argued that dementia cannot be “wiped out” like an infectious disease, and will require a forward-thinking, comprehensive policy change in preventive public health so as to engage with the reality of aging. It is important that different perspectives are taken into consideration so as to avoid “silver bullet” approaches for dementia prevention, as has happened with pharmacological interventions [97]. Just as research into risk factors into dementia requires a focus on health justice, so too should activist research that aims at making society healthier and fairer [98].

What is ultimately required is a situation of shared responsibility between citizens, researchers, and policymakers [99]. The ultimate goal is to “promote individual behavioral change while targeting structures” [65], i.e. encourage behavior change while improving immediate environments and long-term societal constraints in ways that favor brain health. One way of demonstrating this compatibility of simultaneous surface-and-deeper change would be to include deprivation as an explicit risk factor for dementia alongside other behavioral factors, which encourages the provision of support and resources to help people change their lifestyles [36]. This includes social and psychological support, to create an ideal situation in which various actors collaborate to produce motivated, healthy and satisfied citizens [100] within a dementia-resilient society that actively anticipates and struggles against broader determinants of health injustice.

## Funding

This research did not receive any specific grant from funding agencies in the public, commercial, or not-for-profit sectors.

## Declaration of Competing Interest

The authors declare that they have no known competing financial interests or personal relationships that could have appeared to influence the work reported in this paper.
